# Modelling of Tissue Invasion in Epithelial Monolayers

**DOI:** 10.3390/life13020427

**Published:** 2023-02-02

**Authors:** Faris Saad Alsubaie, Hamid Khataee, Zoltan Neufeld

**Affiliations:** 1 School of Mathematics and Physics, The University of Queensland, Brisbane, QLD 4072, Australia; 2 Australian Bureau of Statistics, Brisbane, QLD 4000, Australia

**Keywords:** cell competition, cellular Potts model, invasion, travelling wave

## Abstract

Mathematical and computational models are used to describe biomechanical processes in multicellular systems. Here, we develop a model to analyse how two types of epithelial cell layers interact during tissue invasion depending on their cellular properties, i.e., simulating cancer cells expanding into a region of normal cells. We model the tissue invasion process using the cellular Potts model and implement our two-dimensional computational simulations in the software package CompuCell3D. The model predicts that differences in mechanical properties of cells can lead to tissue invasion, even if the division rates and death rates of the two cell types are the same. We also show how the invasion speed varies depending on the cell division and death rates and the mechanical properties of the cells.

## 1. Introduction

A challenging question in developmental biology and cancer research is how cellular properties influence cell competition in epithelial tissues, which is crucial in normal tissue development and elimination of transformed precancerous cells [1,2,3,4]. Epithelial cell layers line organs throughout the body [5] and play important roles in tissue and organ development, yet account for about 90% of all cancers [6]. In cell competition, normal and mutant cell populations with different properties compete with each other at their interface for survival, where the winner cells invade and occupy the space held by the loser cells, which are eventually eliminated [1,2,3,7]. A key question is how this competition process is influenced by the cellular interactions and properties. Answers to this question can provide new information in the development of cancer treatments and regenerative medicine [2].

Theoretical models are ideal tools to assess the main mechanisms of the competitive cell invasion phenomenon [8]. Various models have been developed to study the cell competition process, including differential equations of predator–prey interactions [7,9], continuous mechanical models of cell proliferation and death [4,10], the vertex model of the evolution of cell turnover and local topology dynamics [11], and the cellular Potts model (CPM) of regulation of mechanical contact-dependent competition [12]. However, it remains elusive how cellular properties affect the speed of cell competition in which winner cells occupy the space following loser cells’ apoptosis.

To fill this gap, we propose a computational model to analyse how different properties of epithelial cells determine the speed of competitive invasion. The model represents the generation and progress of the cell invasion process in a generic epithelial layer due to various differences in the cell parameters. In principle, such differences may arise due to mutations characteristic of cancer cells within the resident population that may lead to invasion of resident cells by the modified cell type. The proposed model is based on the CPM [13], a computational modelling framework that enables the simulation of epithelial cell dynamics (e.g., cell shape and cell–cell interactions), while being computationally and conceptually simpler than most off-lattice models (e.g., vertex model) [14,15,16,17]. The model simulates the competition between two cell populations with different properties. Cell shapes and interactions evolve according to the Potts energy model. Cell proliferation and death events are associated with mechanical properties of cells and modelled as stochastic events. The modelling results are discussed in the context of experiments and other theoretical studies.

This paper is organised as follows. Section 2 presents the development of the model. This section first presents how the dynamics of cellular shapes and interactions between cells are modelled. It also describes modelling cell proliferation and death processes. In Section 3, the computational results are presented and discussed. First we investigate the equilibrium state of a single cell type as a function of the parameters, then we analyse competition between cell types that differ either in their turnover rates or bio-mechanical properties. Finally, results are discussed in context with earlier findings, followed by potential future research directions.

## 2. Model Description

To model the effect of cellular properties on epithelial cell invasion, we propose a two-dimensional computational model that represents the competition of two cell types within an epithelial monolayer. The model is based on the CPM such that cells are represented on a lattice, where each cell covers a set of connected lattice sites (or pixels) and each pixel can only be occupied by one cell at a time.

The expansion and retraction of the cell contours is determined by stochastic minimisation of a phenomenological energy function [15,16,18,19,20,21]:

(1) $$E=\lambda_{\mathrm{area}}\sum_{\alpha =1}^{N}{\left(A_{\alpha }-A_{0}\right)}^{2}+\lambda_{\mathrm{cont}}\sum_{\alpha =1}^{N}L_{\alpha }^{2}+\sum_{\vec{i},\vec{j}}\phi \left(\alpha_{\vec{i}},\alpha_{\vec{j}}\right)\left(1-\delta (\alpha_{\vec{i}},\alpha_{\vec{j}})\right)$$

where $A_{\alpha }$ and $L_{\alpha }$ are the area and perimeter of each cell α, respectively, of *N* cells with indices α = 1, …, N. The first term represents preference of the cells to maintain a preferred area $A_{0}$, and $\lambda_{\mathrm{area}}$ is an area compressibility coefficient, which determines how easily cells can deviate from the preferred area. The second term describes the contractility of the cell perimeter, where the penalty parameter $\lambda_{\mathrm{cont}}$ represents cortical actomyosin contractility around the lateral cell membrane [22]. The last term represents cell–cell adhesion, mediated by adhesion molecules such as E-cadherin [23]. The parameters $\lambda_{\mathrm{area}},\lambda_{\mathrm{cont}}$, and ϕ determine the relative contribution of mechanical properties of the cells to the energy function *E*, and thus to the configuration of cells in the monolayer. $\phi (\alpha_{\vec{i}},\alpha_{\vec{j}})$ is the boundary energy cost at neighbouring lattice sites $\vec{i}$ and $\vec{j}$. The Kronecker δ function prevents counting pixels that belong to the same cell. When both lattice sites $\vec{i}$ and $\vec{j}$ correspond to cells, $\phi \left(\alpha_{\vec{i}},\alpha_{\vec{j}}\right)=\lambda_{\mathrm{adh}}$; otherwise when one or both lattice sites represent boundary walls surrounding the monolayer, the boundary energy cost ϕ is set to zero. Note that $\lambda_{\mathrm{adh}}<0$ to represent that cells preferentially expand their boundaries shared with neighbouring cells. This is, however, balanced by the contractility of the cell perimeter. In this paper, we use Equation (1) to model the effect of mechanical properties of cells on epithelial cell invasion in a two-dimensional space. This model considers a minimal set of parameters efficient for model analysis along with the cell proliferation and death rate parameters. The model can be extended to three-dimensional space for considering further properties, such as extracellular components [24].

A cell expands or shrinks following a stochastic series of elementary steps. The algorithm selects two adjacent lattice sites $\vec{i}$ and $\vec{j}$ that belong to different cells $\alpha_{\vec{i}}\neq \alpha_{\vec{j}}$. Then, it attempts to copy the cell index $\alpha_{\vec{i}}$ into the adjacent lattice site $\vec{j}$, which takes place with probability [25,26]:

(2) $$P\left(\alpha_{\vec{i}}⟶\alpha_{\vec{j}}\right)=\left\{\begin{array}{cc} 1 & \;\Delta E\leq 0\\ e^{-\Delta E/T} & \;\Delta E>0 \end{array}\right.$$

where ΔE is the change of energy in functional (1) due to this elementary step, and the temperature-like parameter *T* is a scaling factor that represents the magnitude of stochastic fluctuations in the model. Following our earlier works [15,16], we set T=50 since it provides cell shapes in both the soft and hard regimes for various combinations of parameters in Equation (1). The time unit of the model is given by a Monte Carlo step (MCS) that corresponds to selecting every pixel site in the lattice once for a copy attempt described above [25]. Taken together, Equations (1) and (2) imply that cell configurations that increase the penalties in functional (1) are less likely to occur. Thus, the population of cells evolves through stochastic rearrangements in accordance with the biological factors incorporated into the effective energy function *E*.

To model the competition for space between two cell populations, it is also necessary to include a turnover of cells through stochastic cell birth and death events [9,12]. A crucial factor that regulates cell proliferation is cell growth factor [27,28], so that the probability of cell proliferation for individual cells increases with the cell area [29]. We therefore define cell proliferation as a stochastic event: at every MCS, a set of uniformly distributed random numbers in [0,1] are generated for each cell that reaches the target area (i.e., $A_{\alpha }\geq A_{0}$). If the random number is smaller than the cell division parameter *B*, the corresponding cell is divided into two cells each with area $\approx A_{0}/2$. Then, according to Equations (1) and (2), these two daughter cells grow and approach the target area $A_{0}$.

We model the cell death process using characteristics identified experimentally. Experiments have shown that during the death process of a cell, the cell cortical actin network, together with microtubule and intermediate filaments, become depolymerised [30,31], and the interactions of the cell with its neighbouring cells become disorganized [32], leading to the degradation of cellular components and the loss of cell volume (i.e., cell shrinkage) [32,33]. Accordingly, in our model, within every MCS, each cell can die with a probability *M*. The parameters of the dead cells are modified by setting the contractility and adhesion parameters to zero (i.e., $\lambda_{\mathrm{cont}}=0$ and $\lambda_{\mathrm{adh}}=0$). Also, the target area of the dead cell is set to zero ($A_{0}=0$), and the area compressibility is increased to $\lambda_{\mathrm{area}}=200$ so that the dead cell gradually shrinks and then disappears from the layer. Using computer simulations, we found that $\lambda_{\mathrm{area}}=200$ was sufficient to avoid the accumulation of a large number of partially compressed dead cells in the system over time.

We note that in a real tissue, cell division and death may be influenced by multiple factors, including signals from surrounding cells and interactions with the extracellular matrix. For simplicity, in this model we do not consider these factors, in order to focus on the main mechanisms determined by the competition for space of two cell types with different properties.

We implement computational simulations of the cell invasion model using the open-source software package CompuCell3D (CC3D) [25]. Each simulation starts with two cell types (each with 225 cells or 900 cells and different set of parameters) placed on a rectangular domain and separated by a barrier. In the initial condition, the area of each cell was set to the target area $A_{0}$, i.e., each cell occupies a square shaped area of 10×10 pixels. The simulation domain is surrounded by wall cells that prevent the cells from sticking to the lattice boundaries. The wall cells are excluded from participating in the pixel copies of the Potts model [34].

We run each simulation in three stages. First, cell death and division events are switched off, and the two cell types are separated by a barrier. In this stage, both cell types reach their equilibrium shapes according to their mechanical parameters in the energy function *E*. As was shown earlier, when the contractility $\lambda_{\mathrm{cont}}$ is high (or the cell–cell adhesion coefficient is low) the cells form quasi-polygonal approximately hexagonal shapes (hard regime) [18,19]. In the opposite case of low contractility, the cell shapes are more irregular, with strongly fluctuating dynamical boundaries (soft regime). This implies that the model takes into account both the shape and size of the cells in representing the invasion process. In the second stage of the cell invasion simulation, both cell death and division are allowed (according to the stochastic rules described above), while the two cell types remain separated by the barrier. During this stage, each cell type reaches their equilibrium density and the corresponding average cell size. The competition between the two cell types takes place in the third stage when the separating barrier is removed. The simulation of this stage is usually continued until one of the cell types disappears from the domain.

## 3. Results and Discussion

In order to calibrate the model parameters to represent a realistic epithelium, first we investigate the equilibrium state of a homogeneous monolayer composed of a single cell type. We analyse how the equilibrium cell density changes with the model parameters, such as the cell division and death probabilities and the mechanical parameters of the CPM energy function.

We then model cell competition with two different spatially separated cell types and examine how one cell type invades the region occupied by the other, depending on the model parameters. First, we will focus on competition between cells that have the same mechanical parameters but different biological properties, represented by the probabilities of cell division and death. Then we consider the case where competing cell types have the same cell turnover parameters but differ in their mechanical properties characterised by parameters in the energy function, such as cortex contractility, area compressibility, and cell–cell adhesion. Then, we will also model the cell competition of two cell types in case they have different biological and mechanical properties at the same time.

### 3.1. Homeostatic Equilibrium in a Homogeneous Cell Layer

To investigate how the model parameters affect the equilibrium cell density, we analyse the behaviour of a cell layer with a single cell type occupying the entire simulation domain. In the initial condition, the area of each cell was set to the target cell area $A_{0}=100$, and the domain size is 200×200 lattice sites, which is initially fully occupied by $N_{0}=400$ cells. The simulation parameters are listed in Table 1. Then, the CPM simulation is run until the total number of cells reaches a stationary state in which there is a dynamical equilibrium between the stochastic cell division and cell death processes. We checked that the domain size and cell numbers are sufficiently large so that the results of the simulations are robust and not affected by random fluctuations of individual cells.

We found that for all parameter values considered, the total number of cells at equilibrium is higher then the initial cell number $N_{0}$, corresponding to cells occupying the target area $A_{0}$. This means that the cells are slightly compressed at equilibrium, i.e., $\langle A_{\sigma }\rangle <A_{0}$, and therefore cell division is suppressed by crowding. However, random cell death events result in apoptotic shrinking cells extruded from the layer. This creates space for the neighbouring cells to grow and reach the target area, so that a cell division can take place in one of the neighbours of the dead cell.

**Figure 1:**
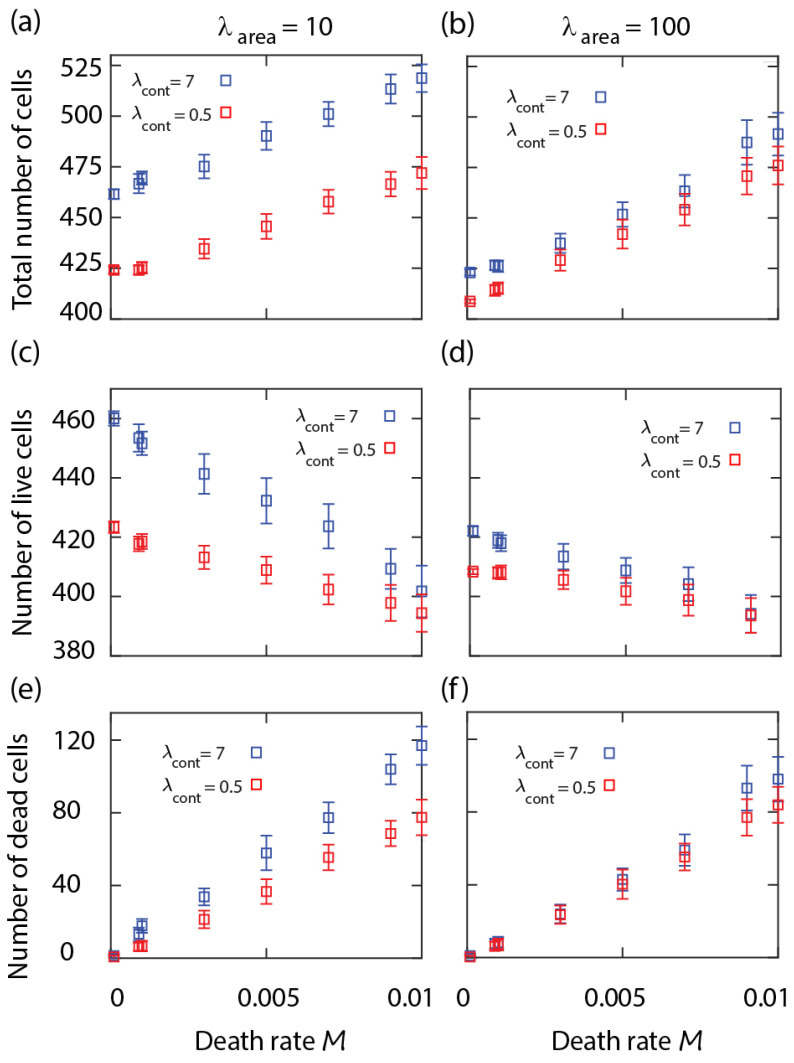
Number of cells at equilibrium in a cell sheet with single cell type versus cell mortality rate *M*. Total number of cells (top row), number of live cells (middle row), and number of dead cells (bottom row) versus *M* at cell area compressibility $\lambda_{\mathrm{area}}=10$ (**a**,**c**,**e**), 100 (**b**,**d**,**f**), and various cell perimeter contractility $\lambda_{\mathrm{cont}}$ (inset). Other simulation parameters: Birth rate B=0.03 and $\lambda_{\mathrm{adh}}=-10$ (see Movies S1–S4). Each symbol is derived from an individual simulation run and corresponds to mean ± SD calculated over time in the stationary state.

We studied the parameter dependence of the equilibrium cell density. The numerical results show that the total number of cells at equilibrium increases with the cell mortality rate *M*; see Figure 1a. However, this is caused by the increasing number of dead cells within the layer that shrink over time and disappear, while the number of live cells decreases slightly with the death rate. The overall number of both live and dead cells is higher when the cell perimeter is more contractile (i.e., $\lambda_{\mathrm{cont}}=7$); see Figure 1a–c. Increasing cortex contractility, $\lambda_{\mathrm{cont}}$, reduces stochastic fluctuations at the cell boundaries [15]. Therefore, in the neighbourhood of dead cells, the process of elimination of the dying cell becomes slower. In contrast, when live cells have softer boundaries (i.e., $\lambda_{\mathrm{cont}}=0.5$), they are more likely to change their shape and advance into the region occupied by a shrinking dead cell. This effect is more clear at lower area strain $\lambda_{\mathrm{area}}$, where the cells area $A_{\alpha }$ can deviate more from the target cell area $A_{0}$. According to Equations (1) and (2), with strengthening $\lambda_{\mathrm{area}}$, the cell area approaches $A_{0}$ more closely, slowing down the shrinkage of the dead cells; compare Figure 1a,c,e with Figure 1b,d,f.

**Figure 2:**
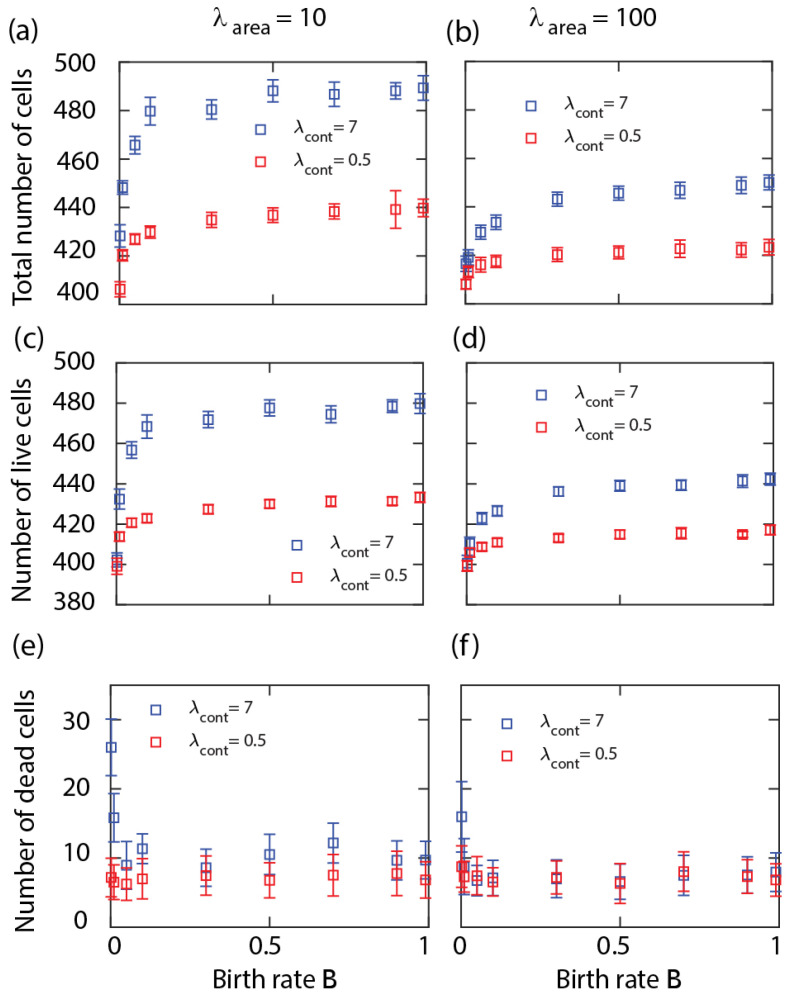
Number of cells at equilibrium in a cell sheet with single cell type versus cell birth rate *B*. Total number of cells (top row), number of live cells (middle row), and number of dead cells (bottom row) versus *B* at cell area compressibility $\lambda_{\mathrm{area}}=10$ (**a**,**c**,**e**), 100 (**b**,**d**,**f**), and various cell perimeter contractility $\lambda_{\mathrm{cont}}$ (inset). Other simulation parameters: Death rate M=0.001 and $\lambda_{\mathrm{adh}}=-10$. Each symbol is derived from an individual simulation run and corresponds to mean ± SD.

We also examined the variation of equilibrium cell density by altering the cell proliferation probability *B*. At small values of *B*, the equilibrium number of cells increases sharply and then saturates from around B≈0.1; see Figure 2a,c,d. Thus, the density is mostly unaffected by the cell division probability except when *B* is small. Note that in the B→0 limit, there is no well-defined equilibrium between the birth and death processes so the change around a small *B* may be due to very long transients. Similarly to the results in Figure 1, the cell density increases with the cell perimeter contractility; see Figure 2a–c. This is explained by the slower shrinkage of the dead cells surrounded by live cells with hard boundaries. Results in Figure 2d–f show that the cell density decreases slightly with the area compressibility $\lambda_{\mathrm{area}}$.

From these simulation results, we see that the proportion of the dead cells within the cell layer increases with the death rate parameter *M*, is relatively insensitive to the birth rate, and changes slightly with the mechanical parameters. In order to limit the proportion of dead cells within a realistic range for a biologically functional epithelial cell layer (e.g., well below 10%), in the following cell competition simulations, we will restrict the range of the death rate parameter to 0<M≤0.003; see Figure 1e,f.

### 3.2. Competitive Invasion due to Different Division and Death Rates

**Figure 3:**
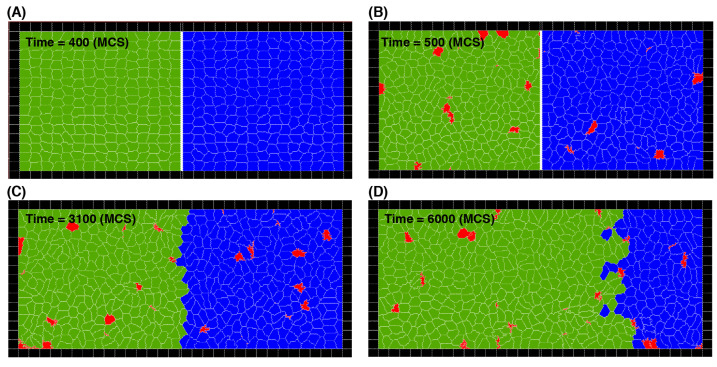
Three stages of the simulations of the cell invasion process. (**A**) Stage 1: cell division and death are switched off for both cell types (green and blue) to allow cells to reach their shape at mechanical equilibrium. Cell types are separated by a barrier (white). (**B**) Stage 2: cell division and death are switched on to allow cells to reach their steady density and size for both types, while separated by the barrier. Red cells indicate dead cells, which gradually shrink and disappear. (**C**,**D**) Stage 3: the separating barrier is removed, and the green cell type invades the region of the blue cell type, because in the case shown, the green cells have a greater division probability (B=0.9) than the blue cells (B=0.1), while the death rates are the same ($M_{1}=M_{2}=0.003$). The invasion is shown at two different time points (**C**,**D**). For simulation movies, see Movies S6–S8.

Next, we model competitive cell invasion with two cell types that initially occupy the left and right halves of the domain. This follows the three simulation stages outlined in Section 2; see Figure 3. Briefly, first, a simulation is run to reach equilibrium of cell shapes within the two sub-domains separated by a barrier. Then, cell proliferation and death are enabled to reach equilibrium cell density and size for each cell type separately. Finally, the barrier is removed, and the two cell types compete for space.

To analyse the dynamics of the simulated competitive cell invasion, we first examine the effects of cell division and death rates. We consider cell types that have the same mechanical parameters, but they are different in either the cell division or death probabilities. A series of snapshots of typical cell distributions is shown in Figure 3 where green and blue represent different cell types (in this example green cells have higher division probability) and red represents dead cells.

**Figure 4:**
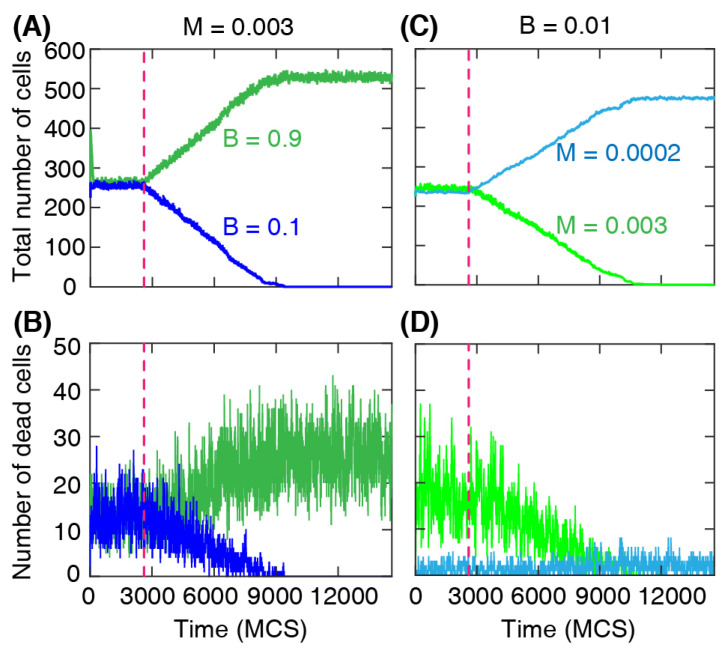
Total number of cells and number of dead cells for each cell type over time during the invasion. The mechanical properties are the same for both types: $\lambda_{\mathrm{area}}=70$, $\lambda_{\mathrm{cont}}=7$, and $\lambda_{\mathrm{adh}}=-10$. (**A**,**B**) The death probability M=0.003 is the same for all cells. Cells with greater division probability (B=0.9) invade the region occupied by cells with lower division probability (B=0.1). (**C**,**D**) When cell division probabilities are the same (B=0.001), the cells with a lower death rate (M=0.0002) invade the region occupied by cells with a greater death rate (M=0.003). The invasion is demonstrated by the approximately linear increase of the number of invading cells (**A**,**C**). Dashed line: time of wall removal.

The changes in the cell numbers over time for each type are shown in Figure 4A,B. After the barrier removal, the number of dominant cells increases approximately linearly, while at the same time the number of the other cell type decreases and eventually reaches zero. At that stage, the whole domain is fully occupied by the dominant cell type only. We find a similar change in the number of cells when the cells have different death rates; see Figure 4C,D. The simulation shows a well-defined linear regime in the numbers of each cell type during the invasion process, indicating that the front separating the invading and invaded cells progresses with an approximately constant speed.

We analysed the dependence of the invasion speed on the division and death rate parameters. The simulation parameters are shown in Table 2. First the slope of the linear growth of the total number of invading cells over time was determined, then the invasion speed was calculated as the average rate of change of the number of invading cells divided by the equilibrium density ρ corresponding to the parameters of the invading cells, which was estimated earlier from the simulations with a single cell type:

(3) $$v=\frac{\Delta N}{\Delta t}\frac{L}{\rho }$$

**Figure 5:**
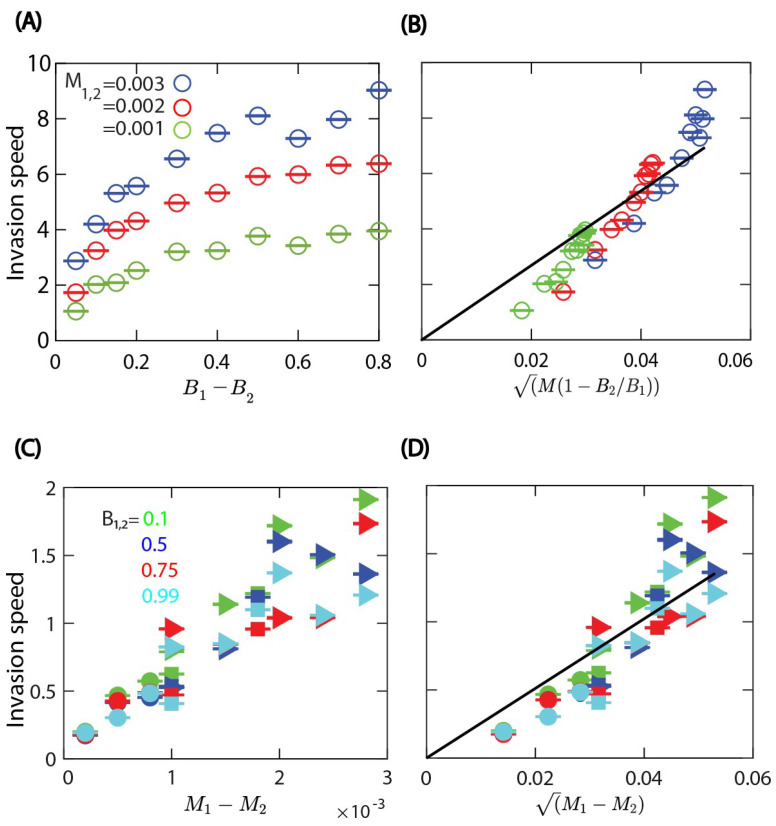
Cell invasion speed changes with differences in the cell division and death probabilities, when the mechanical properties are the same for both cell types: $\lambda_{\mathrm{area}}=70$, $\lambda_{\mathrm{cont}}=7$, and $\lambda_{\mathrm{adh}}=-10$. (**A**) The invasion speed increases with the difference in cell division probabilities. The division rate parameter of cell type 2 is set to $B_{2}=0.1$. Both cell types have equal death probabilities (see color legend). (**B**) Comparison of the invasion speed with the functional form predicted by the reaction–diffusion model when the cell death probabilities *M* are the same; see Equation (7). Black line: linear fit y(x)=134.37x. (**C**) The invasion speed increases with the difference in death probabilities when both types have equal proliferation probability $B_{1}=B_{2}=0.1$. (**D**) Invasion speed versus the square root of the difference of death probabilities; see Equation (8). Black line: linear fit y(x)=25.55x. Colors represent different birth rates (see color legend), and the symbols indicate the death rate of the first cell type, i.e., $M_{1}=0.001$ (circle), $M_{1}=0.002$ (square), and $M_{1}=0.003$ (triangle). In (**A**–**D**), open symbols: cell type 1 invades the area occupied by cell type 2; closed symbols: cell type 2 invades the area occupied by cell type 1. Each symbol is derived from an individual simulation run and corresponds to mean ± SD.

We chose the width of the domain perpendicular to the direction of invasion as the length unit, L=1. When one of the rates is the same for both cell types, we find that the invasion speed increases with the difference between either the cell division or death rates; see Figure 5A–D. As expected, results in Figure 5 show that the invading cell type is the one with the higher division rate or the lower death rate.

To interpret the numerical results obtained for the invasion velocity shown in Figure 5, we compare the behavior of the CPM model to an analogous reaction–diffusion model with two competing species described by the following system of partial differential equations [35]:

(4) $$\begin{array}{c} \frac{\partial \rho_{1}}{\partial t}=b_{1}\rho_{1}\left(1-\rho_{1}-\rho_{2}\right)-m_{1}\rho_{1}+D\nabla^{2}\rho_{1}\\ \frac{\partial \rho_{2}}{\partial t}=b_{2}\rho_{2}\left(1-\rho_{1}-\rho_{2}\right)-m_{2}\rho_{2}+D\nabla^{2}\rho_{2} \end{array}$$

where $\rho_{1}\left(r,t\right)$ and $\rho_{2}\left(r,t\right)$ are the densities of the competing species, $b_{1}$ and $b_{2}$ are the reproduction rate constants, $m_{1}$ and $m_{2}$ are the mortality parameters, and *D* is the diffusion coefficient. The spatially uniform system has three steady states: (0,0), $\left(0,1-m_{2}/b_{2}\right)$, and $\left(1-m_{1}/b_{1},0\right)$. Note that in general, there is no co-existence of the two species in the uniform system (except in the special case when $b_{1}/m_{1}=b_{2}/m_{2}$). Linear stability analysis of the uniform steady states shows that (0,0) is unstable and, of the remaining two states, one is stable and the other is unstable. For the case when $b_{1}/m_{1}>b_{2}/m_{2}$, the first species is dominant and the stable steady state is $\left(1-m_{1}/b_{1},0\right)$, while the other steady state is unstable. In the opposite case, the second species dominates, and the only stable uniform steady state is $\left(0,1-m_{2}/b_{2}\right)$.

When the initial condition of the reaction–diffusion model is such that the two species are spatially separated, a Fisher–Kolmogorov type travelling wave solution develops, where the stable state corresponding to the dominant species propagates into the space occupied by the other species: $\rho_{1,2}\left(r,t\right)=f_{1,2}\left(x-vt\right)$, where *v* is the front velocity moving with constant speed along the *x* axis. For this type of so-called “pulled” front, where the stable phase is pulled into the unstable phase by the instability at the leading edge, the front velocity is determined by the linear instability near the unstable state [35,36,37]. For concreteness, let us assume that the first species dominates, i.e., $b_{1}/m_{1}>b_{2}/m_{2}$. Thus, in the neighbourhood of the leading edge, the density of the invading species is small, while the second species is close to the unstable equilibrium: $\rho_{1}\ll 1,\rho_{2}\approx 1-m_{2}/b_{2}$. Therefore, the reaction–diffusion equation can be linearised as:

(5) $$\frac{\partial \rho_{1}}{\partial t}=\rho_{1}\left(m_{2}\frac{b_{1}}{b_{2}}-m_{1}\right)+D\nabla^{2}\rho_{1}.$$

Note that this equation is equivalent to the analogous linear approximation of the standard Fisher–Kolmogorov travelling wave problem [38], and the corresponding asymptotic wave velocity (for sufficiently sharp initial conditions, i.e., spatially separated cell types) is:

(6) $$v=2\sqrt{D\frac{b_{1}m_{2}-b_{2}m_{1}}{b_{2}}}.$$

In the special case, when $m_{1}=m_{2}=m$, the formula simplifies to:

(7) $$v=\sqrt{D\frac{m(b_{1}-b_{2})}{b_{1}}}$$

or in the other case, with equal division rates $b_{1}=b_{2}=b$, we have

(8) $$v=\sqrt{D(m_{2}-m_{1})}.$$

Although the continuous reaction–diffusion model and the CPM represent quite different modelling approaches, the results for the invasion front speed *v* in Equations (6)–(8) are qualitatively consistent with our numerical results obtained from the CPM simulations. First, in the case of equal death rates $M_{1}=M_{2}$, we find that the invasion front speed increases with the difference between the cell division rates and also increases with the common death rate; see Figure 5A. The rescaled data in Figure 5B show that the invasion speed is an increasing function of $M(1-B_{2}/B1)$ as in Equation (7). Then, in the case of equal cell division rates $B_{1}=B_{2}$, we find that the invasion front velocity increases with the difference in the death rates and is not affected much by the common cell division rate; see Figure 5C,D. The functional form appears to be consistent with $v∼\sqrt{M_{1}-M_{2}}$.

We note that the CPM model does not have a simple parameter for cell motility that can be linked to the diffusion coefficient of the PDE model. This may account for the limitations of the similarity between the theoretical prediction based on the reaction–diffusion model and the simulations, since random cell motility in the CPM may depend on multiple parameters. We also note that the inherent fluctuations in the stochastic CPM model seem to play a role in the somewhat irregular variability of the front velocity shown in Figure 5. Of course, it might be possible to reduce the fluctuations by using a larger domain size with more cells; however, this leads to a much higher computational cost of the simulation.

### 3.3. Invasion Due to Different Mechanical Properties

To test whether differences in mechanical properties of the cells alone can also generate invasion waves, we run simulations with cell types that have the same biological parameters (i.e., equal death and division rates) but different values for the mechanical parameters in the energy function of the CPM in Equation (1).

**Figure 6:**
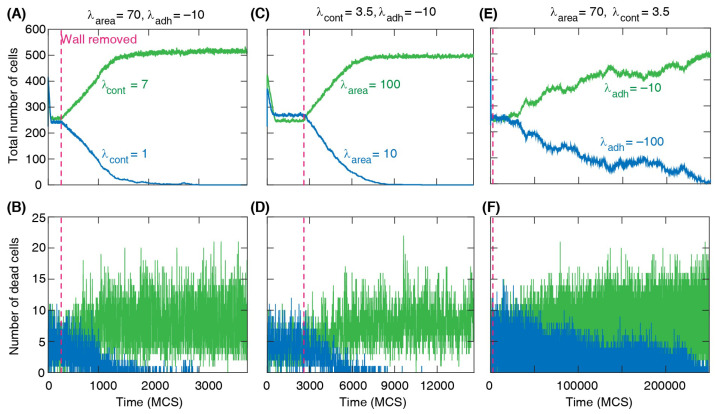
Total number of cells and number of dead cells vs time for competing cell types with different mechanical properties, where division and death probabilities are the same for all cells, B=0.99 and M=0.001. (**A**,**B**) Cells with stronger perimeter contractility ($\lambda_{\mathrm{cont}}=7$) invade the region occupied by cells with softer shapes ($\lambda_{\mathrm{cont}}=1$). The area elasticity $\lambda_{\mathrm{area}}=70$ and cell-cell adhesion $\lambda_{\mathrm{adh}}=-10$ are the same for all cells. (**C**,**D**) With fixed $\lambda_{\mathrm{cont}}=1$ and $\lambda_{\mathrm{adh}}=-10$, cells with greater area elasticity ($\lambda_{\mathrm{area}}$ = 100) invade the region occupied by cells with lower area elasticity ($\lambda_{\mathrm{area}}$ = 10). (**E**,**F**) With fixed $\lambda_{\mathrm{area}}=70$ and $\lambda_{\mathrm{cont}}=3.5$, cells with weaker adhesion ($\lambda_{\mathrm{adh}}=-10$) invade the region occupied by more adhesive cells ($\lambda_{\mathrm{adh}}=-100$). (**A**,**C**,**E**) The invasion is demonstrated by the linear increase in the total number of invading cells. (**A**–**F**) Dashed line: time of wall removal.

Through tracking the number of cells over time, results show that cells with higher perimeter contractility $\lambda_{\mathrm{cont}}$ invade softer cells with lower contractility; see Figure 6A,B. Similarly, higher area compressibility parameter $\lambda_{\mathrm{area}}$ of cells leads to invasion when all other parameters are the same for both cell types; see Figure 6C,D. In the case of cell–cell adhesion strength, we found that cells with a weaker cell–cell adhesion invade more adhesive cells; see Figure 6E,F. However, in this case, the invasion speed is much lower compared to the previous cases, indicating a weaker competitive advantage of cells with modified cell–cell adhesion.

**Figure 7:**
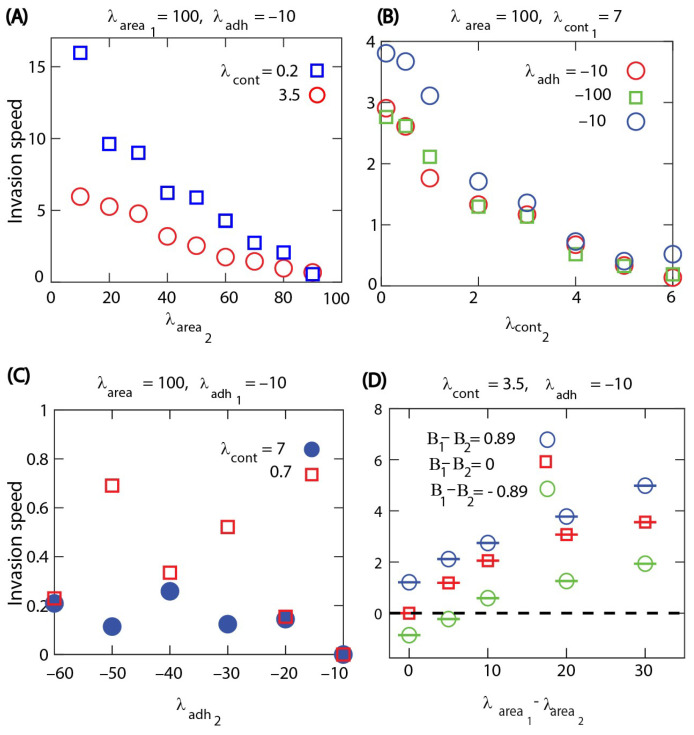
Cell invasion speed changes with variations in the mechanical properties and the biological properties of cells, where the cell division and death probabilities are the same for both cell types at B=0.99 and M=0.001 in (**A**–**C**). (**A**) Invasion speed versus cell area elasticity $\lambda_{\mathrm{area}}$ at different cell contractilities $\lambda_{\mathrm{cont}}$. (**B**) Invasion speed versus $\lambda_{\mathrm{cont}}$ at different cell–cell adhesions $\lambda_{\mathrm{adh}}$. In (**B**), we also tested the invasion velocity when we have higher death rates for both cell types such as $M_{1}=M_{2}=0.003$ (blue circle). (**C**) Invasion speed versus $\lambda_{\mathrm{adh}}$ at different $\lambda_{\mathrm{cont}}$. (**D**) Invasion speed versus the differences of cell area elasticities of the two cell types at different division rates of the cells. In (**A**–**C**), open symbols: cell type 1 invades the area occupied by cell type 2. Closed symbols: cell type 2 invades the area occupied by cell type 1.

We then studied how the invasion speed changes with the mechanical properties of competing cell types. The invasion speed is calculated as the slope of the linear increase of the total number of invading cells divided by the equilibrium density. When the invasion is due to different area compressibility, the invasion speed increases monotonically with the difference in the area compressibility. The numerical results also show that the invasion is faster when both cell types are softer, i.e., having a weak cortex contractility; see Figure 7A. When the contractility of the cells are different, the invasion speed increases with the difference in the contractility parameters; however, it appears to be unaffected by changing the common area compressibility; see Figure 7B. In this case, we also found that the invasion speed increases with the cell death probability. This is consistent with the role of cell turnover in the cell invasion process. Even when the cell invasion is due to different mechanical properties, the turnover of cells by death and division is necessary for competitive cell invasion. Increasing the cell death probability speeds up the turnover of both types of cells, which then contributes to faster invasion. In the case with different cell–cell adhesion parameters, we see that the invasion speed is much smaller than in the previous cases and is only slightly affected by cell contractility; see Figure 7C. The numerical results in Figure 7C also show that contractility may influence the direction of the invasion. In the case of hard cells with high contractility such as $\lambda_{{\mathrm{cont}}_{1,2}}=7$, cell type 2, i.e., the more adhesive cell type, invades cell type 1, while the invasion direction changes to the opposite when both cell types are less contractile soft cells with $\lambda_{{\mathrm{cont}}_{1,2}}=0.7$.

Finally, we also studied the competitive cell invasion in the case when there are differences in both biological and biomechanical parameters at the same time. Figure 7D shows how these combined differences can enhance the invasion speed when the cell type with a higher compressibility parameter also has a higher division rate. In the opposite case, when the division rate of the cell type with a higher compressibility parameter is reduced, the invasion becomes much slower or the direction of invasion may change. Thus, biomechanical differences can compensate for the higher division rate of a competing cell type and could be used to control invading cells.

## 4. Discussion

In the simulations presented here, we gained some insights into how cellular properties may influence the cell competition process. Our modelling results predict that differences in cell properties can generate invasion waves at the interface of two types of cells. The invasion front moves with a constant speed, and the invasion is generally faster when the differences between the parameters of the two cell types are larger.

In the case of a cell layer with a single cell type, when there is a dynamic equilibrium between cell division and cell death events, we found that the cell density is always higher than the neutral density corresponding to cells with target area $A_{0}$. Therefore, the cell division is suppressed almost everywhere except in the neighbourhood of cell death events where live cells can expand, reach the cell division threshold, and then replace the lost cell.

We can obtain a lower bound for the average equilibrium cell size, or upper bound for cell density. For the live neighbouring cells to be able to reach the threshold $A_{0}$ requires that $A_{\alpha }+A_{\alpha }/N_{c}\geq A_{0}$, where $N_{c}$ is the average cell coordination number, i.e., the number of live cells surrounding a dead cell. In the hard cell boundary regime (i.e., with a large contractility parameter), the cell layer forms a hexagonal honeycomb type structure with $N_{c}=6$ [19]. For lower contractility, the shape of the cells will change; however, the average number of neighbouring cells can be expected to remain similar. Thus we obtain a lower bound for the average cell size in equilibrium as $A_{\alpha }=A_{0}N_{c}/\left(1+N_{c}\right)\approx 5A_{0}/6.$ This approximation is in agreement with the numerical results in Figure 1 and Figure 2, showing that when starting the simulations with 400 cells with their area equal to $A_{0}$, at equilibrium the total number of live cells is bounded from above by N≈400×6/5 = 480.

In the cell competition process at the interface between two cell types, the different cell properties result in a biased competition for replacing the missing cell after cell death. For example, when there is a difference in cell death rates, one cell type is more likely to generate some extra space at the boundary, but the neighbouring cells have an equal chance of replacing the missing cell. When the cell death rates are the same but cell division probabilities are different, the probability of replacing the dead cell is biased towards the cells with a higher division rate. This is essentially the same mechanism that leads to the invasion waves in simple lattice models (e.g., [39]) where each competitor is represented by a single lattice site. This mechanism, however, cannot explain the invasion wave induced by mechanical differences in the two cell types, and such differences cannot be represented in simple stochastic lattice models of competing populations [35,39].

The computational study presented here provides experimentally testable predictions for the process of competitive cell invasion. Although some of the model parameters are not easily accessible and modifiable in experiments, the regulation of cell cortex contractility by multiple signaling pathways may provide an option for investigating competitive cell invasion and comparing it with the predictions of our model. For our simulations, we used the computational modelling framework of the CPM. It would be interesting to test whether the same behaviour can be observed with alternative multicellular modelling approaches, e.g., the vertex model. Mechanical cell competition was also studied in simplified one-dimensional elastic spring models [10]. This type of reduced model may allow for developing analytical approaches to gain better insights into the mechanisms that determine the characteristics of cell invasion.

Here we focused on the average rate of invasion in cell competition. However, fluctuations and variability along the front interface can also be important in some biological or medical applications. Such fluctuations were studied using simple lattice models [35,39] and could be extended further multicellular models.

## Figures and Tables

**Table 1:** Figure 1 parameters.

Parameter	Value
Domain size	200×200 pixels
Initial cells size	100 pixels
$\lambda_{\mathrm{area}}$	100, 10
$\lambda_{\mathrm{cont}}$	7, 0.5
$\lambda_{\mathrm{adh}}$	−10
$A_{0}$	100 pixels
Number of cells	400
*T*	50
*B*	0.03
*M*	Variable

**Table 2:** Figure 5 parameters.

Parameter	Value
Domain size	600×300 pixels
Initial cells size	100 pixels
$\lambda_{\mathrm{area}}$	70
$\lambda_{\mathrm{cont}}$	7
$\lambda_{\mathrm{adh}}$	−10
$A_{0}$	100 pixels
Number of cells	1800
*T*	50
*B*	Variable
*M*	Variable

## Data Availability

The model codes used in the numerical simulations are available on request from the corresponding author.

## Supplementary Materials

The following supporting information can be downloaded at: https://www.mdpi.com/article/10.3390/life13020427/s1, Movie captions, Movie S1: A simulation of a monolayer with a single cell type (green). Simulation parameters are: B=0.03, M=0.003, $\lambda_{\mathrm{area}}=10$, $\lambda_{\mathrm{adh}}=-10$, and low contractility $\lambda_{\mathrm{cont}}=0.5$ corresponding to “soft” cells. Red cells: dead cells that gradually shrink and disappear, Movie S2: Monolayer with a single cell type (green) in the "hard" regime. Simulation parameters are: B=0.03, M=0.003, $\lambda_{\mathrm{area}}=10$, $\lambda_{\mathrm{adh}}=-10$, and high contractility corresponding to hard cells $\lambda_{\mathrm{cont}}=7$, Movie S3: A simulation of a monolayer with a single cell type (green) with high area strain and low contractility. Simulation parameters are: B=0.03, M=0.003, $\lambda_{\mathrm{area}}=100$, $\lambda_{\mathrm{cont}}=0.5$, and $\lambda_{\mathrm{adh}}=-10$, Movie S4: A simulation of a monolayer with a single cell type (green) with high area strain parameter and high contractility. Simulation parameters are: B=0.03, M=0.003, $\lambda_{\mathrm{area}}=100$, $\lambda_{\mathrm{cont}}=7$, and $\lambda_{\mathrm{adh}}=-10$, Movie S5: A simulation of a monolayer with two cell types. Simulation parameters are: B=0.9, M=0.001, $\lambda_{\mathrm{area}}=70$, $\lambda_{\mathrm{cont}}=7$, and $\lambda_{\mathrm{adh}}=-10$ (green); B=0.1, M=0.001, $\lambda_{\mathrm{area}}=70$, $\lambda_{\mathrm{cont}}=7$, and $\lambda_{\mathrm{adh}}=-10$ (blue), Movie S6: A simulation of a monolayer with two cell types. Simulation parameters are: B=0.99, M=0.007, $\lambda_{\mathrm{area}}=100$, $\lambda_{\mathrm{cont}}=0.2$, and $\lambda_{\mathrm{adh}}=-10$ (green); B=0.99, M=0.001, $\lambda_{\mathrm{area}}=10$, $\lambda_{\mathrm{cont}}=0.2$, and $\lambda_{\mathrm{adh}}=-10$ (blue), Movie S7: A simulation of a monolayer with two cell types. Simulation parameters are: B=0.99, M=0.002, $\lambda_{\mathrm{area}}=100$, $\lambda_{\mathrm{cont}}=7$, and $\lambda_{\mathrm{adh}}=-10$ (green); B=0.99, M=0.002, $\lambda_{\mathrm{area}}=100$, $\lambda_{\mathrm{cont}}=1$, and $\lambda_{\mathrm{adh}}=-10$ (blue), Movie S8: A simulation of a monolayer with two cell types. Simulation parameters are: B=0.99, M=0.001, $\lambda_{\mathrm{area}}=70$, $\lambda_{\mathrm{cont}}=0.7$, and $\lambda_{\mathrm{adh}}=-10$ (green); B=0.99, M=0.001, $\lambda_{\mathrm{area}}=70$, $\lambda_{\mathrm{cont}}=0.7$, and $\lambda_{\mathrm{adh}}=-60$ (blue).
